# Measurement of Stress Waves Propagation in Percussive Drilling

**DOI:** 10.3390/s21113677

**Published:** 2021-05-25

**Authors:** Diego Scaccabarozzi, Bortolino Saggin

**Affiliations:** Mechanical Department, Politecnico di Milano, Polo Territoriale di Lecco, 23900 Lecco, Italy; bortolino.saggin@polimi.it

**Keywords:** stress wave propagation, percussive drilling, drill bit, strain gauges, chatter vibration

## Abstract

This work describes the results of a test campaign aimed to measure the propagation of longitudinal, torsional, and flexural stress waves on a drill bit during percussive rock drilling. Although the stress wave propagation during percussive drilling has been extensively modeled and studied in the literature, its experimental characterization is poorly documented and generally limited to the detection of the longitudinal stress waves. The activity was performed under continuous drilling while varying three parameters, the type of concrete, the operator feeding force, and the drilling hammer rotational speed. It was found that axial stress wave frequencies and spectral amplitudes depend on the investigated parameters. Moreover, a relevant coupling between axial and torsional vibrations was evidenced, while negligible contribution was found from the bending modes. A finite element model of the drill bit and percussive element was developed to simulate the impact and the coupling between axial and torsional vibrations. A strong correlation was found between computed and measured axial stress spectra, but additional studies are required to achieve a satisfactory agreement between the measured and the simulated torque vibrations.

## 1. Introduction

The mining and construction industries exploit percussive drilling to bore and fragment natural rock or concrete. Due to the combined action of drilling and thrust, a high rate of penetration can be achieved. Percussive drilling breaks rock using consecutive blows aimed to create a crack through the worked material while rotation removes the fragmented part. At each blow, stress wave propagation occurs through the drill bit. The phenomenon has been well understood and described by analytic modeling in the case of simple and homogeneous geometry [1], i.e., cylindrical bars or rods, but its explanation becomes difficult in most of the practical applications, where real geometries and constraint conditions are present.

The mechanics of percussive drilling have been addressed in the literature [2,3,4,5,6] through analytical, numerical, and experimental studies aimed to clarify energy conversion, transfer, and efficiency. The interaction between the worked material and the drill bit depends on the force penetration curve, which allows the development of numerical models [7] to predict the efficiency of the percussive drilling. The force-penetration curve is generally defined by one [8] or two strain gauges [7,9] mounted at sections far from the impact region. The interaction between the drill bit and the worked material has also been modeled with lumped parameters modeling three-dimensional finite element methods [10,11,12,13,14]. The available studies provide useful information about the resistance during the impact and the efficiency of the energy transfer [15,16], but do not consider the drill bit rotation and the matching between axial and torsional vibrations, i.e., the chatter vibration phenomenon [17].

Moreover, even referring to recent studies [18,19,20], the main findings are the design and modeling of the percussive systems, with little testing activity related to the percussion efficiency characterization. The measurement of the stress wave propagation during percussive drilling is poorly documented, and no information is present about the torsional and flexural torques experienced by the drill bit during the drilling.

With the generated stress waves being the main contribution to the penetration of the drill bit during the working, the characterization of their frequency content would provide useful information for the design of the percussive system and the related penetration efficiency. Moreover, the tracking of these characteristics in the frequency domain would allow the improvement of the modeling of the interaction between the drill bit and the worked material, which is another open topic to increase the efficiency of the percussive drilling systems.

Thus, this work aims to provide a complete characterization of the stress waves propagation on a drill bit commonly used for rock demolition, measuring the axial, torsional, and bending waves generated during percussive drilling. Tests were performed while varying three parameters at two different levels, the drilling speed, the feeding force, and the worked material. This allowed the study of the changes in the forcing frequency and stresses during the drilling due to the interaction between the drill bit and the material. Experimental results have been explained using a FE model, developed to simulate one blow impact.

The paper is organized as follows. Section 2 describes the adopted measurement procedure, setup, and FE model. Section 3 provides experimental activity results and a comparison between the experiments and the simulations. Results are discussed in Section 4, and the paper is eventually concluded in Section 5.

## 2. Materials and Methods

### 2.1. Measurement Procedure and Setup Description

The characterization of the stress propagation inside the drill requires several measurements. The main stress components are bending torque, torsional torque, and axial forces. Measurements are provided by the application of strain gauges on the drill bit (model Speed X 520 by Bosch), thus characterizing the whole state of stress at one point of the tool. Stress propagation has been studied by simulating common working conditions. The experimental campaign is based on three parameters: the rotary speed of the bit, the feeding force exerted by the user, and the mechanical properties of the perforated material. The testing matrix covers two levels for each parameter, two rotary speeds, two levels of the feeding force, and two classes of resistance for the concrete. The design of the experiment was based on three parameters, two levels full factorial scheme with eight testing conditions:

- The drill bit rotational speed was set to 22.3 and 29.3 rad/s; hereafter, the numbers 4 and 6 identify the low and high levels of the rotary speed;
- The feeding force provided by the operator was set to 100 N and 200 N; a variability of about 30% of the nominal force was tolerated;
- LC 25/26 and C 45/55 concrete types were tested, whose resistance classes are defined according to the European concrete standard EN 206-1.

Table 1 summarizes the testing matrix.

Each test was marked by a three digits identification number (IDN); the code can be read starting from the left as follows:

- The first digit is 0 if the tested material is LC 25/26;
- The second digit is 1 in the case of the lowest feeding force;
- The third digit is 0 in the case of the lowest rotational speed.

As an example, code 110 describes testing on C 45/55 material, feeding force at 100 N, and rotational speed 4.

**Figure 1:**
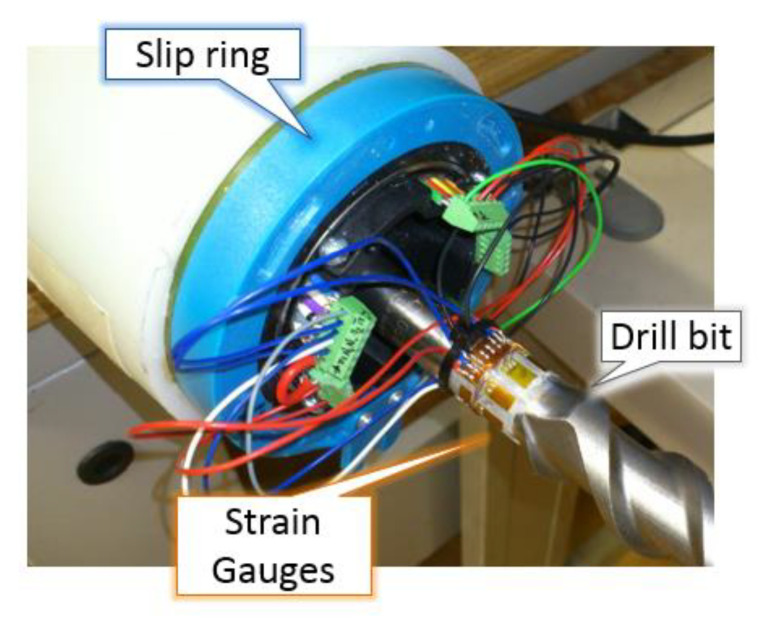
Strain gauges mounting on the drill bit and cables connected to the slip ring system.

Strain gauges were mounted on the cylindrical part of the drill bit at a distance of about 10 cm from the impact surface. Axial stress was measured by EA-06-120LZ-120/E rectangular strain gauges mounted in a full-bridge configuration; torque was measured with HBM rosettes, 1-XY41-1.5/120 type. Flexural stresses during the working were detected using EA-06-120LZ-120/E rectangular strain gauges mounted in a half-bridge configuration. Epoxy glue X60 was used to tie strain gauges to the drill bit. A Vishay 2310 conditioning amplifier provided both the conditioning and the amplification of the strain gauges’ signals. The amplifier consists of ten independent units, each including a stabilized voltage generator. Four slots were used for the conditioning of the bridges. Each unit can manage either a full or a half-bridge, and in the latter case, the amplifier allows the completion of the bridge configuration by adding two internal dummy resistors. The conditioning unit decreases the measured signal of 0.5 dB at 25 kHz and 3 dB at 65 kHz. To assure the connection with the rotating drill bit, a slip ring has been used to provide a continuous signal transfer between the static and rotary side of the percussion driller (GBH7 Bosch type GBH 7-46 DE) either from the strain gauges or the amplifiers. The slip ring is a type SC 104 manufactured by LTN Servotechnik GmbH. Figure 1 shows a view of the strain gauge mounting and the drill bit with the slip ring used for the experimental activity.

**Figure 2:**
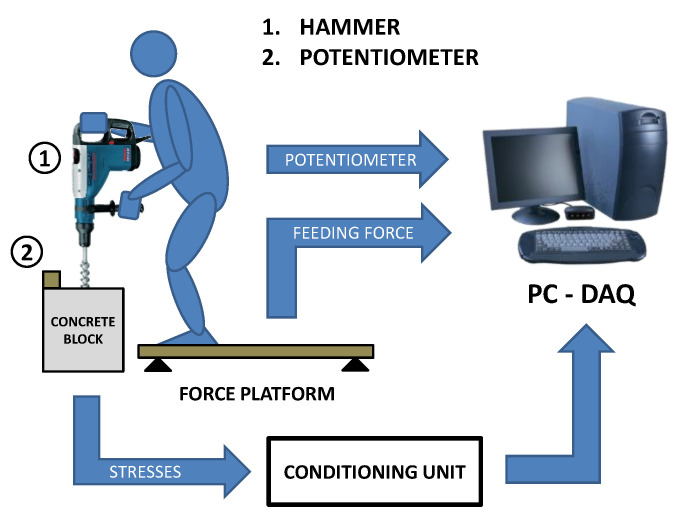
Scheme of the measurement setup.

In addition to the strain measurements, the operator weight was collected utilizing a platform equipped with three HBM load cells type Z6FD1, conditioned and amplified by HBM Scout 55. Load cell sensitivity is 10 mVN^−1^ and has an full input scale of 1000 N. The feeding force is therefore indirectly measured by the change of the operator weight during the drilling. Moreover, hole depth was extracted from the hammer displacement measurement performed by a string potentiometer (Celesco SP1-12) that was attached between the concrete block and the hammer. The potentiometer has a maximum stroke of 317 mm and nominal sensitivity of 31.5 mVmm^−1^. Figure 2 shows a scheme of the testing setup and highlights the measurement chain.

Tracking of the operator force and the penetration depth was deemed necessary to establish if a relationship exists between the stress wave amplitudes during working and the measured parameters.

Two independent measurement systems were used for the acquisition; one was based on a NI 9234 acquisition board with a sampling rate set at 2 kHz, the second one used a NI 9215 board with a sampling frequency of 40 kHz. The lowest sampling rate was used to measure the feeding force and the penetration depth. In order to avoid misleading results caused by the first phases of perforation and assure repeatability of the testing, an initial bore of about 1 cm was made on the tested concrete samples, and then drilling was completed once at least 5 cm depth was reached.

### 2.2. Impact Simulation

In order to simulate the stress wave propagation during the impact, a finite element model of the drill bit has been developed using the commercial software Abaqus 3DS Simulia. The geometry of the striker has been simplified, retaining only the geometry of the beating surface, but a dummy mass was added to it to match the actual kinetic energy before the impact. The latter modification was possible because the striker is more rigid than the drill bit.

**Figure 3:**
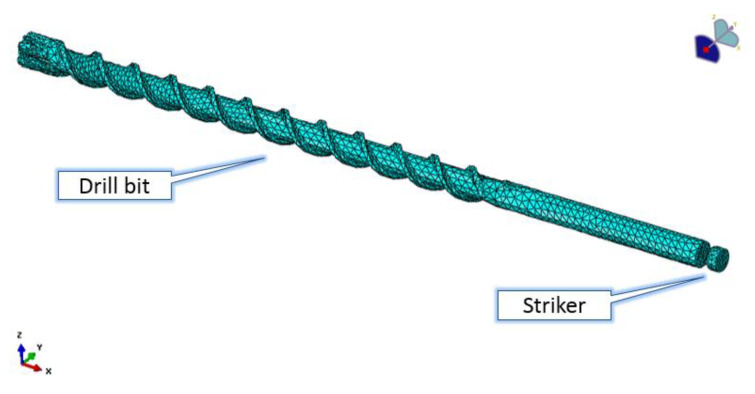
FE model of the drill bit with the striker.

De-featuring was performed on the drill bit as well since the carbide inserts were removed. Figure 3 shows the 3D finite element model of the drill bit with the striker. AISI 4140 was used for the drill bit material. The material density, the Poisson’s coefficient, and the elastic modulus were set to 7850 kgm^−3^, 0.29 and 205,000 MPa, respectively. The latter values refer to the drill bit material characteristics. Mesh was made of four-node linear tetrahedral elements, 14,479 and 2864 for the drill bit and the striker, respectively, to model the complex geometry of the drill bit flute, and these were validated by the software tools to check for elements of distortion and shape factor. The drill bit model was partitioned at the position where strain gauges were mounted, and the stresses during the simulated impact were computed at that position. Sampling time was set to 25 µs, and a numerical antialias filter was applied to the simulation results. Explicit integration has been performed at two steps; the first step applied a speed of 10 ms^−1^ to the striker along the drilling direction, while the second step computed the drill bit dynamics after the impact. The overall simulated time was 0.022 s.

In order to match the expected shifting of the forcing frequency due to the interaction of the drill bit with the worked material, the drill bit was connected to the ground through an axial spring and dashpots. The properties of the added elements were tuned to minimize the error between the measured and computed frequencies and spectral amplitudes.

**Figure 4:**
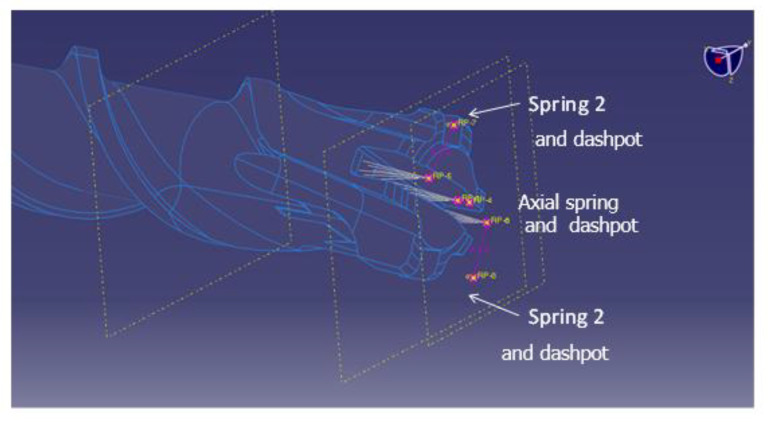
Inclined stiffnesses and dashpots to model the coupling between axial and torsional vibrations.

Coupling between the torsional and axial vibration during the hammering has been modelled, adding two inclined springs and dashpots at the drill bit connection to the ground, as shown in Figure 4, which shows a detailed view of the added elements at the end of the drill bit.

Other than the elastic and damping parameters, the inclination and the connected area between the elements and the drill bit were varied to minimize the difference between measured and computed torque spectra. This required an additional partition of the drill bit, varying the diameter of the surface at which the drill bit end is connected with the simulated springs and dashpots.

## 3. Results

### 3.1. Strain Gauges Calibration

Scale factors of the strain gauges were analytically determined using the known elastic properties and geometry of the drill bit and the gain factor introduced in the measurement chain by the conditioning unit. The latter was derived by a shunt resistance calibration performed with the signal conditioning unit. Analytical sensitivities were compared to the ones derived by experimental calibration with reference loads, which were obtained from the weight of calibrated masses. The axial bridge was calibrated with a PCB 086C02 dynamometric hammer with 11.2 mVN^−1^ sensitivity, 444 N full scale, and 1% linearity. Table 2 provides a comparison between experimental and analytical scale factors.

The analytical sensitivity uncertainty was computed, propagating [21] the error contribution of each parameter involved in the analytical sensitivity computation. As an example, sensitivity for the axial force measurement “*s*” can be derived as:

(1) $$s=\frac{E_{0}}{2\;A\;E}G\;k_{f}\left(1+\upsilon \right),$$

where *E*_0_ is the bridge supply voltage, *G* is the amplification gain, *k_f_* is the strain gauge sensitivity, *A* is the drill bit area at the strain gauges attachment, and *E* and *υ* are the elastic modulus and the Poisson’s coefficient of the drill bit material, respectively. Uncertainty about the axial force sensitivity was then derived as:

(2) $$u_{s}=\sqrt{{\left(\frac{\partial s}{\partial E_{0}}u_{E_{0}}\right)}^{2}+{\left(\frac{\partial s}{\partial A}u_{A}\right)}^{2}+{\left(\frac{\partial s}{\partial k_{f}}u_{k_{f}}\right)}^{2}+{\left(\frac{\partial s}{\partial \upsilon }u_{\upsilon }\right)}^{2}+{\left(\frac{\partial s}{\partial G}u_{G}\right)}^{2}+{\left(\frac{\partial s}{\partial E}u_{E}\right)}^{2}},$$

where *u_E_*_0_, *u_A_*, *u_kf_*, *u_υ_*, *u_G_* and *u_E_* are the uncertainties of the bridge supply voltage, drill bit area, strain gauge sensitivity, material Poisson’s coefficient, amplifier gain, and material elastic modulus, respectively.

Propagation gave relative uncertainties for the analytical sensitivities ranging between 3% and 3.5%. The relative uncertainty of the measurement chain sensitivities obtained from the experimental calibration was found to be within 2–2.5%, suggesting compatibility between the analytically predicted sensitivities and the experimental ones.

Thus, the measured sensitivities were finally used to scale the measured voltage from the applied strain gauges during the test campaign.

### 3.2. FE Model Validation

Drill bit vibration modes were measured in unconstrained conditions. The drill bit was suspended on elastic wires and excited along the drilling direction using a dynamometric hammer while the strain gauges measured the drill bit response due to the excitation. A Polytec laser Doppler vibrometer OFV-505 measured the drill bit response as well. This was performed to allow partial validation of the strain gauge measurements. Table 3 provides a comparison between the measured free axial vibration modes with the strain gauges, the laser Doppler vibrometer, and the ones computed with the FE model.

**Figure 5:**
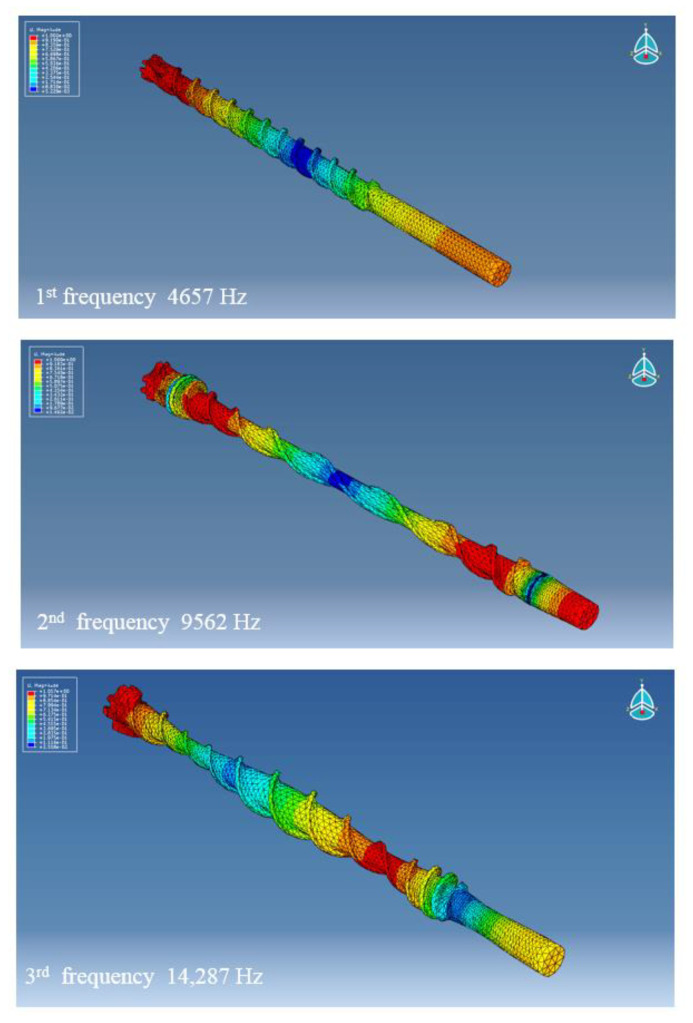
The first three axial modes of vibration for the drill bit FE model.

The computed modes of vibration are reported in Figure 5.

### 3.3. Stress Waves Measurements

**Figure 6:**
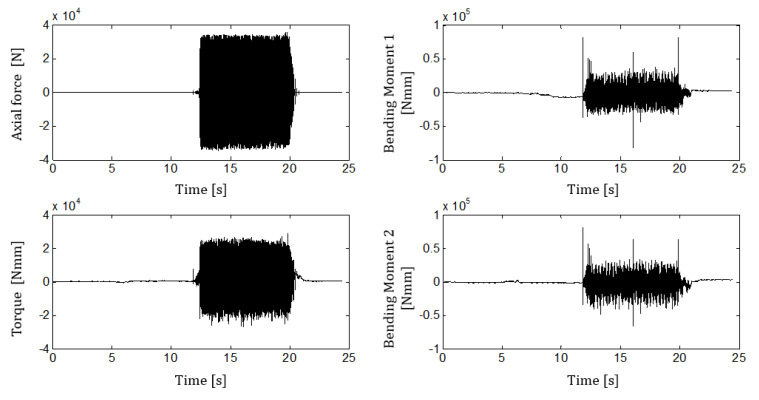
Measured time histories with strain gauges, test case IDN 020.

**Figure 7:**
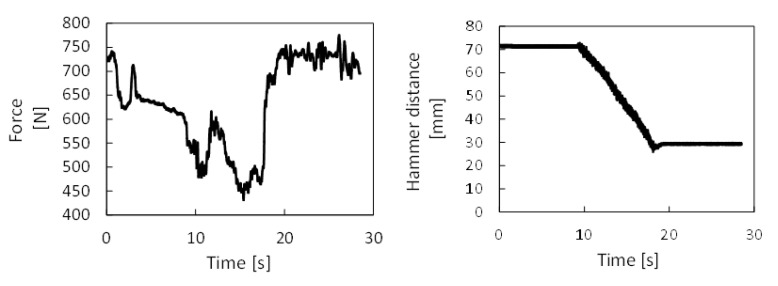
Measured time histories for the hammer displacement and the operator’s weight, test case IDN 020.

Figure 6 shows measured forces, torque, and bending moments for one case (test 020), while in Figure 7, the drilling depth and feeding force are provided. The time scales are different because the signals were acquired using two independent acquisition systems.

**Figure 8:**
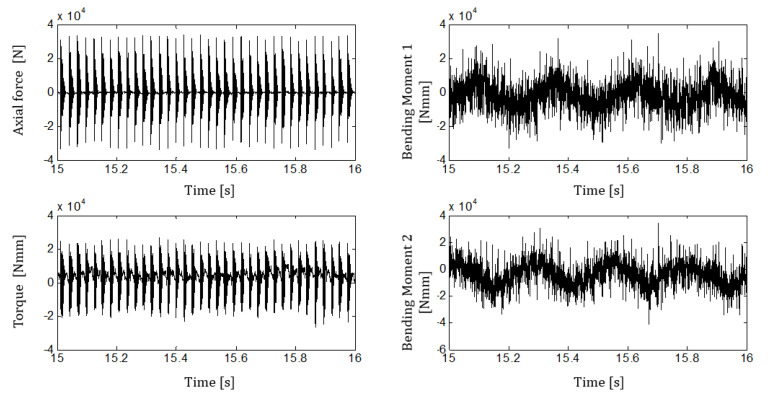
Time zoom of the measured force and torques, test IDN 020.

Figure 8 provides a detailed view of the measured force, torque, and bending moments. In order to evaluate the measurement repeatability, a portion of the acquired signals with constant feeding force was extracted. Axial force and torque signals were triggered, taking 100 blows and 1024 points for each blow. For each buffer point, the maximum, the minimum, and the root mean square (RMS) of the measured signals were extracted. The statistics about the measured force and torques for test 020 are reported in Table 4.

**Figure 9:**
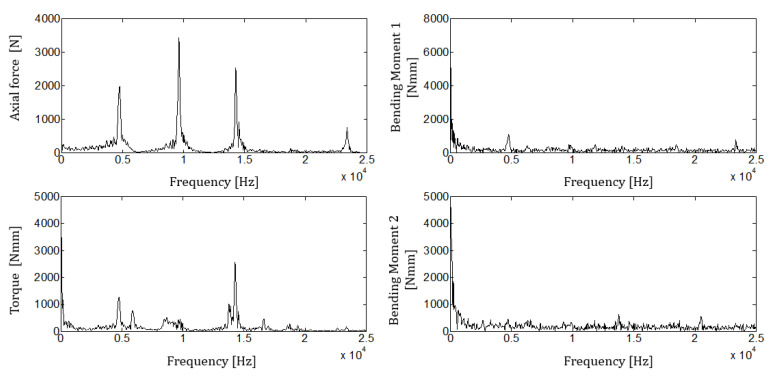
Axial force and torques spectra of a single blow, test IDN 020.

The measured axial forces, bending momenta, and torques were analyzed to compute their spectra. Results for a single blow are shown in Figure 9 (test IDN 020), whereas average axial force and torque spectral amplitudes, computed from triggered signals extracted in a time frame where the feeding force was constant, are provided in Table 5 and Table 6, as well as the measurements repeatability for the overall testing matrix. f_1_, f_2_, and f_3_ refer to the first three frequencies where the spectral amplitudes of the axial and torsional stress waves are maximized. A_1_, A_2_, and A_3_ are the measured maximum spectral amplitudes for the axial and torsional waves. The relative standard deviations, provided in Table 5 and Table 6, which are related to the measured spectral amplitudes and frequencies, have been computed as the ratio between the measured standard deviation and the average.

The measured average axial forces were statistically analyzed to highlight if measured eigenfrequencies and related amplitudes depend on the experimental factors. The considered parameters are the investigated ones, the rotational speed, the operator feeding force, and the worked material. Results of the multi-factor ANOVA analyses are reported in Table 7, where it can be highlighted that the chosen parameters affect the measured spectral amplitudes and frequencies for the axial stress waves.

### 3.4. FE Results

**Figure 10:**
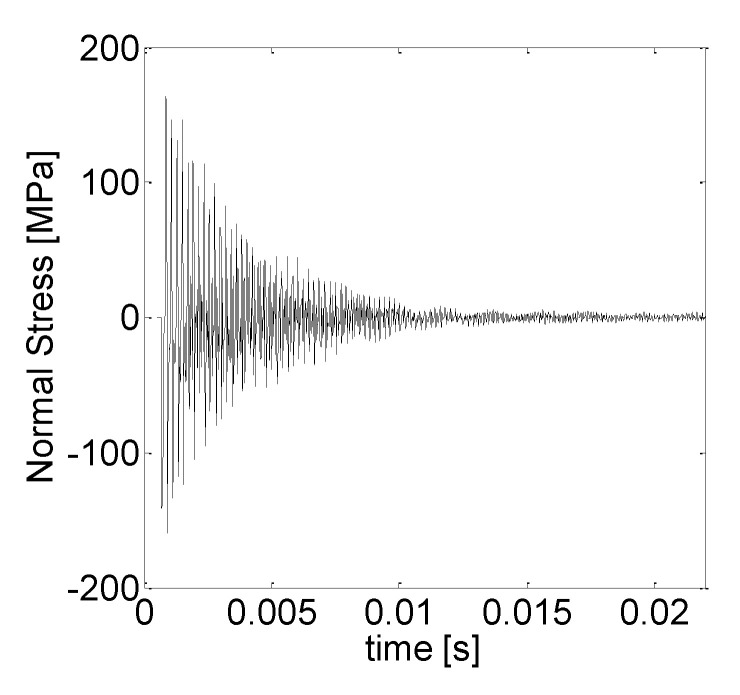
Time history of the computed axial stress.

**Figure 11:**
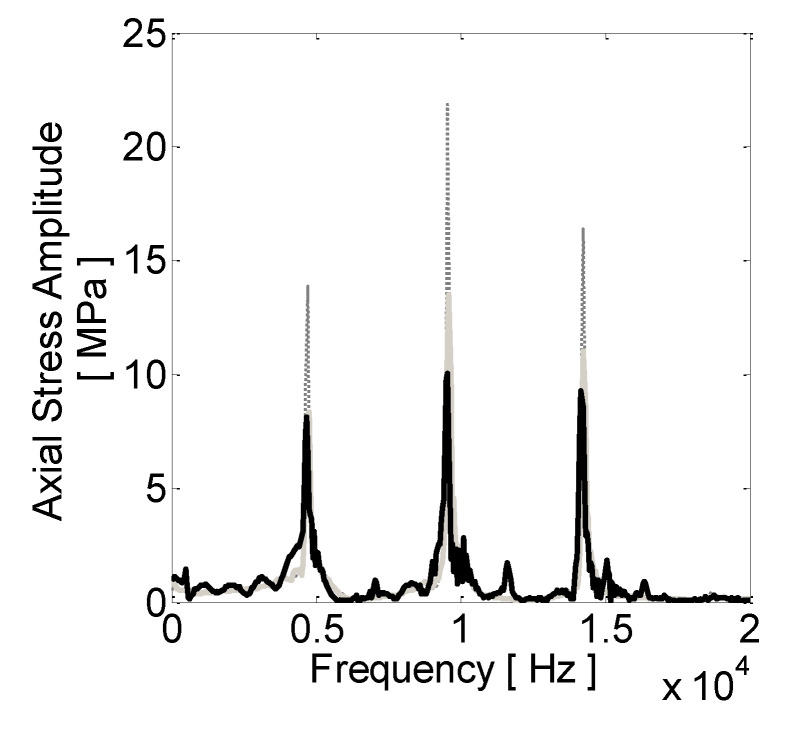
Measured and computed spectra for the axial stress wave. Black, light grey, and dashed dark grey show computed axial stress by means of the FE model and the ones measured in tests 020 and 010, respectively.

Figure 10 provides the computed time history of the stress generated by a simulated impact, whereas Figure 11 shows average axial spectra for the tests IDN 020 and 010 and the spectrum computed by the model.

**Figure 12:**
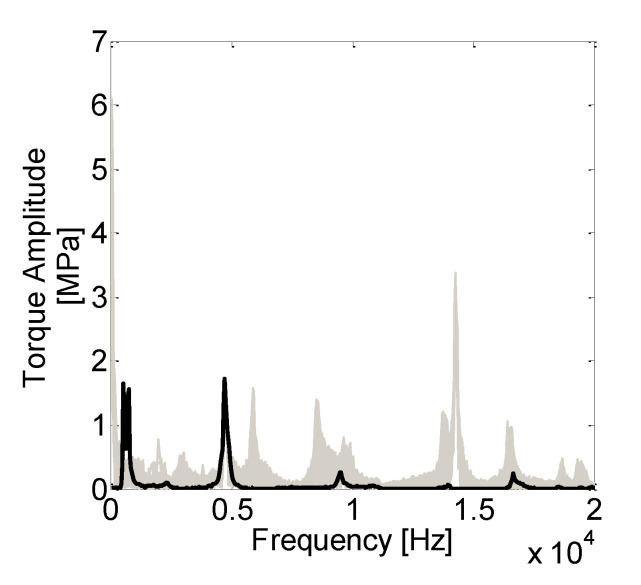
Measured and computed spectra for the torsional stress wave. Blackline shows computed spectrum while light grey curves provide the measured spectra for the test IDN 020.

Spectral amplitudes and frequencies for the axial stress waves are summarized in Table 8. The optimal values of the elastic and damping properties were found to be 10 kNmm^−1^ and 0.25 Nmm^−1^s, respectively, for the inclined stiffnesses and dashpots and 20 kNmm^−1^ and 0.25 Nmm^−1^s for the axial ones, respectively. The optimized values for the inclination angle and acting diameter were 8° and 16 mm, respectively. Figure 12 provides a comparison between the measured and computed spectra for the torsional stress wave.

## 4. Discussion

A strong agreement was found between strain gauge measurements and vibrometer readings. As shown in Table 3, the error of the drill bit’s axial frequencies is less than 0.05%. Moreover, the error between the computed and measured frequencies is lower than 0.7% for the FE model. The obtained result was considered acceptable, especially if compared with the expected measurement uncertainty, set to about 2% for the used measurement chain.

Concerning the measurements during drilling (as shown in Figure 6 and Figure 8), the strain gauge’s time histories provided repeatable measurements without signal saturation. Operator feeding force showed small variability during the drilling, from 10 to 20, of about 10% of the nominal value. This was valid for the test IDN 020, but in general, the measured variability increased up to 30%. A difficult control of the feeding force was expected since the high vibration levels during the hammering affect the operator’s posture.

The measured statistics in Table 4 highlights that the axial force and the torque are generally stable. This is demonstrated by the obtained measurement repeatability that for 100 consecutive blows, is around 10%. Repeatability worsens for the bending momenta, ranging between 20% and 42%. The result is clearly shown in Figure 8, where the measured bending torques seem to be modulated by a low-frequency forcing. This result is explained by the impossibility of the operator to keep a perfect vertical alignment during the drilling and is anyway expected as a result of the high vibration levels during the working.

The average measured spectra in Figure 9 confirm the previous result. The force and torque spectra have some components up to 25 kHz, while the bending torques show contributions mainly at a low-frequency range. The axial stress wave frequencies increase by about 1% with respect to the free constraint condition, and coupling between axial and torsional vibration is evidenced, mainly at the first and third measured frequencies. The latter result is somehow similar to the chatter vibrations, a typical phenomenon evidenced in the metal drilling [17].

Axial force and torque spectra repeatability were investigated in more detail for all tested cases, as shown in Table 5 and Table 6. Repeatability of the spectral amplitudes, computed as the relative standard deviation of the measured average values of Table 5 and Table 6 in the same conditions, was found to range between 10% and 30% for the axial force and between 20% and 40% for the torque. Moreover, the spectral amplitudes seem to be dependent on the operator feeding force and the tested material. In particular, the average amplitudes for CL 25/26 material were larger than the ones measured with C45/55 concrete.

ANOVA analyses, whose results are summarized in Table 7, confirmed that measured amplitudes and frequencies are strongly dependent on the feeding force, the worked material, and the drilling speed. Moreover, except for some cases, the combination of these parameters affects the measured quantities as well. Thus, a reasonable conclusion of the performed study seems that if one wants to model the frequency shifting caused by the drilling, an equivalent impedance that would match the experimental results has to be identified on the basis of these three parameters.

The FE model results confirmed the need for adjusting the constraint impedance about the testing case, i.e., the impedance should account for the particular worked material, grip force, and rotational speed. This is highlighted in Figure 11, where the comparison between the computed axial stress and the ones derived from two testing conditions (ID 010 and 020) shows good agreement in only one case, test ID 020. In that case, matching with the experimental results was found to be within 1% for the forcing frequency, while the error in the computed spectral amplitude reached a maximum of 25% for the second forcing component.

The coupling between axial and torsional vibration was achieved in the FE model only for the first frequency (as shown in Figure 12), providing a frequency error of about 2% and amplitude mismatch of about 5%. Moreover, some spectral components around 400 and 500 Hz appeared in the computed spectrum whose presence was justified by torsional contributions added by the simulated inclined stiffnesses and not present in the real measurements.

Thus, the numerical simulations evidenced that the modeling of the interaction between the drill bit and the worked material with linear lumped parameters is not generally effective. Frequency characterization of the interaction between the axial and torsional waves should be carried out, and a more complex impedance as a generalized frequency function should be implemented.

It has to be highlighted that the described results are related to two types of concretes and the used drill bit model, a typical solution for masonry application. Extension of the described research would be welcome, such as applying the described measurement procedure and analyses to different scenarios, e.g., considering the mining industry. This would allow the creation of an extended database to be exploited by drill bit manufacturers aiming to increase the working efficiency and therefore enhance the product added value.

## 5. Conclusions

Complete characterization of the stress wave propagation was performed during percussive drilling on different working conditions, varying the drill rotational speed, the operator feeding force, and the type of worked material. Results showed that the main forcing components in the drill bit derive from the axial force and the torque, while the stresses introduced by dynamic bending torques have minor importance. Moreover, a strong coupling between axial and torsional vibration was detected. This result is new and remarkable because in the literature, the experimental characterization of the three stress waves under real working conditions has never been performed before.

Statistical analyses showed that the spectral amplitude repeatability of the axial force and torque with constant feeding force is within 10–40% for both of the tested materials. Analyses also showed that the forcing frequencies and related spectral amplitudes are strongly dependent on the tested parameters and their interactions.

The knowledge of the stress waves and their couplings allows the improvement of the drill bit design with a more accurate determination of the maximum stresses and their time evolution through FE modeling.

A FE model of the drill bit provided an accuracy comparable with tests repeatability and showed a good matching between the computed and measured spectral amplitudes of the axial stress wave. Model tuning was obtained only in one tested configuration, stressing the need to define a proper equivalent connection for each testing condition. A first attempt to represent the coupling between axial and torsional vibrations using linear mechanical lumped elements showed that only a small part of the measured spectrum could be correctly retrieved. Thus, the interaction between the axial and torsional waves should be characterized with respect to the frequency, and more complex elements such as frequency-dependent impedances should be implemented. The expected evolution of this study would be the parameterization of the model with respect to the characteristics of the tested material to predict the stress waves through a drill bit without the need for testing in actual working conditions. This step would require, along with the numerical model update, a more extended test campaign on different materials aimed to develop a proper database so that a statistical parametric model of the bit-material interaction can be generated.

## Figures and Tables

**Table 1:** Testing matrix table.

Test IDN	Type of Concrete	Feeding Force	Speed Code *
020	LC 25/28	200 N	4
021	LC 25/28	200 N	6
010	LC 25/28	100 N	4
011	LC 25/28	100 N	6
120	C 45/55	200 N	4
121	C 45/55	200 N	6
110	C 45/55	100 N	4
111	C 45/55	100 N	6

* Note: speed codes 4 and 6 refer to 22.3 and 29.3 rad/s rotational speeds of the driller, respectively.

**Table 2:** Strain gauge calibration.

Nomenclature	Bridge Configuration	Measured Quantity	Experimental Scale Factor	Analytical Scale Factor	Units
Bending Torque 1	Half-bridge	Bending torque	157,326	163,455	N mm V^−1^
Bending Torque 2	Half-bridge	Bending torque	160,020	165,725	N mm V^−1^
Axial Force	Full-bridge	Axial force	47,062	49,695	N V^−1^
Torque	Full-bridge	Torsional torque	142,971	134,980	N mm V^−1^

**Table 3:** Comparison between measured and computed axial modes.

Mode of Vibration	Laser Doppler Vibrometer [Hz]	Strain Gauges [Hz]	FE Model [Hz]	FE Model Error %
First	4688	4690	4657	−0.71
Second	9558	9559	9562	0.03
Third	14,235	14,236	14,287	0.36

**Table 4:** Averages and standard deviations (STDs) of maximum, minimum, and RMS values of 100 blows in test IDN 020.

Quantity	Axial Force [N]	Torque [Nmm]	Bending Moment 1 [Nmm]	Bending Moment 2 [Mmm]
Average maximum	32,618	23,779	20,957	19,665
STD maximum	1305	1426	7754	8259
Average minimum	−30,938	−18,843	−23,304	−24,590
STD minimum	2165	2449	7457	7868
Average RMS	6313	7201	7023	7456
Std RMS	378	648	1474	2684

**Table 5:** Measured frequencies and amplitudes for the axial force.

Test idn	Speed Code	Feeding Force	f1	f1 Relative Standard Deviation	A1	A1Relative Standard Deviation	f2	f2 Relative Standard Deviation	A2	A2 Relative Standard Deviation	f3	f3 Relative Standard Deviation	A3	A3 Relative Standard Deviation
		[N]	[Hz]	[%]	[N]	[%]	[Hz]	[%]	[N]	[%]	[Hz]	[%]	[N]	[%]
020	4	250	4735	0.5	2134	12	9607	0.2	3388	19	14,264	0.0	2746	10
021	6	150	4746	0.5	3090	21	9604	0.2	5359	21	14,266	0.1	4186	18
010	4	100	4707	0.2	3471	19	9570	0.2	5527	21	14,263	0.0	4075	14
011	6	80	4755	0.2	3300	24	9610	0.1	6180	16	14,264	0.0	4146	18
120	4	180	4755	0.2	2230	20	9600	0.2	3408	19	14,266	0.1	3439	15
121	6	140	4760	0.6	2345	29	9606	0.2	4235	28	14,270	0.1	4217	21
110	4	95	4732	0.6	3105	42	9581	0.3	5042	31	14,264	0.1	4461	17
111	6	110	4750	0.5	2840	28	9595	0.2	4477	21	14,264	0.0	5126	17

**Table 6:** Measured frequencies and amplitude for the torque.

Test idn	Speed Code	Feeding Force	f1	f1 Relative Standard Deviation	A1	A1Relative Standard Deviation	f2	f2 Relative Standard Deviation	A2	A2 Relative Standard Deviation	f3	f3 Relative Standard Deviation	A3	A3 Relative Standard Deviation
		[N]	[Hz]	[%]	[N]	[%]	[Hz]	[%]	[N]	[%]	[Hz]	[%]	[N]	[%]
020	4	250	4736	0.6	1339	12	9663	1.1	579	21	14,264	0.0	2820	11
021	6	150	4747	0.4	1677	21	9580	0.9	818	25	14,267	0.1	4144	18
010	4	100	4707	0.2	2140	19	9577	1.5	568	25	14,258	0.1	4212	14
011	6	80	4755	0.3	1771	26	9537	1.3	774	24	14,265	0.0	4228	19
120	4	180	4756	0.4	1168	21	9581	1.4	442	33	14,265	0.0	3378	17
121	6	140	4766	0.8	1270	26	9514	1.7	657	33	14,270	0.1	4109	22
110	4	95	4735	0.7	164	39	9549	1.3	61	27	14,263	0.1	433	18
111	6	110	4750	0.5	1531	28	9519	1.4	584	25	14,264	0.0	5010	18

**Table 7:** ANOVA analyses for the axial forces, test IDN 020. In bold the *p*-values lower than 0.05, selected as significance threshold, are evidenced.

Factor	f1	A1	f2	A2	f3	A3
Speed (A)	**0.00**	**0.00**	**0.00**	**0.00**	**0.00**	**0.00**
Force (B)	**0.00**	**0.00**	**0.00**	**0.00**	**0.00**	**0.00**
Material (C)	**0.00**	**0.00**	0.14	**0.00**	**0.01**	**0.00**
Interaction A*B	**0.00**	**0.00**	**0.00**	**0.00**	**0.03**	**0.00**
Interaction B*C	**0.01**	0.40	1.00	**0.00**	0.07	**0.00**
Interaction A*C	**0.00**	**0.00**	**0.00**	**0.00**	0.84	0.73
Interaction A*B*C	**0.00**	**0.00**	**0.00**	0.81	0.17	**0.00**

**Table 8:** Comparison between measured and computed axial frequencies and relative spectral amplitudes. Measured values refer to tests IDNs 020 and 010.

Mode Number	Measured Frequency Test IDN020	Measured Frequency Test IDN 010	FEM Frequency	Axial Stress Spectral Amplitude Test IDN 020	Axial Stress Spectral Amplitude Test IDN 010	FEM Axial Stress Spectral Amplitude
	[Hz]	[Hz]	[Hz]	[MPa]	[MPa]	[MPa]
First	4735	4707	4681	8.34	13.88	8.15
Second	9607	9570	9544	13.5	21.86	10.05
Third	14,264	14,263	14,180	11.04	16.36	9.26

## Data Availability

The data presented in this study are available on request from the corresponding author.
